# Application of Sodium Selenite in the Prevention and Treatment of Cancers

**DOI:** 10.3390/cells6040039

**Published:** 2017-10-24

**Authors:** Marek Kieliszek, Boguslaw Lipinski, Stanisław Błażejak

**Affiliations:** Faculty of Food Sciences, Department of Biotechnology, Microbiology and Food Evaluation, Warsaw University of Life Sciences—SGGW, Nowoursynowska 159 C, 02-776 Warsaw, Poland; stanislaw_blazejak@sggw.pl

**Keywords:** selenium, cancer, fibrin, parafibrin, polymer, blood

## Abstract

Selenium is an essential trace element that occurs in nature, in both inorganic and organic forms. This element participates in numerous biochemical processes, including antioxidant potential, but the mechanism of its anti-cancer action is still not well known. It should be noted that the anti-cancer properties of selenium depends on its chemical form, therapeutic doses, and the tumor type. Higher nutritional doses of selenium can stimulate human immune system. There are several hypotheses concerning the anticancer activity of selenium, including oxidation of sulfhydryl groups in proteins causing their conformational alterations. Conformational changes in proteins have the ability to weaken the activity of enzymes involved in the metabolism of cancer cells. In case of human fibrinogen sodium selenite, but not selenate, it inhibits protein disulfide exchange reactions, thus preventing formation of a hydrophobic polymer termed parafibrin, circulatory accumulation, of which is associated with numerous degenerative diseases. Parafibrin can specifically form a protein coat around tumor cells that is completely resistant to degradation induced with lymphocyte protease. In this way, cancer cells become protected against destruction by the organism’s immune system. Other possible mechanisms of anticancer action of selenium are being still investigated.

## 1. Introduction

Over 50 years ago, the US President Richard Nixon initiated a “war” against cancer. Although billions of dollars have been spent since that time, and great efforts have been taken in this war, cancer is still present, and except a few forms of cancer (e.g., leukemia in children) morbidity and mortality rates remain at the same level. It should be emphasized that a number of pharmacological compounds have been discovered in recent decades, which unfortunately do not cure cancer in humans, but only prolong their lives, and are very expensive and concurrently beneficial to large pharmaceutical companies [1]. Therefore, the convenient belief that cancer is incurable is prevailing. Despite this pessimistic conclusion, scientists continuously focus on different strategies for treating cancer. While chemical drugs destroy both cancer and healthy cells, it turned out that humans have a very efficient immune system that detects and effectively removes cancer cells [2]. The damage of immune system is one of the potential cause of cancer occurrence. Why a majority of solid tumors are resistant to the treatment of cellular immune system, the so-called *immune therapy*, remains a mystery. A number of hypotheses have tried to explain this phenomenon, but none of them have drawn a conclusion on how to avoid and overcome this [3]. This article presents the views on these issues based on decades of research on blood proteins and on our personal experience with selenium and its multidirectional anticancer activity.

Sodium selenite is incorrectly considered to be an antioxidant, by contrast to other forms of selenium. In this article, it is argued that this compound plays an important role at the initial phase of carcinogenesis. A less recognized, albeit even more essential role of selenite is in its stimulation of the cellular immune system [4]. In addition, certain studies indicate that selenite may inhibit angiogenesis, and help to repair the damaged DNA fragments. However, the most important function of this compound in the fighting of cancer may be the direct activation of natural killer (NK) cells. Moreover, and quite unexpectedly, sodium selenite was shown to oxidize sulfhydryl groups on the tumor cell membranes to corresponding disulfides making the unavailable to the exchange with other plasma proteins.

## 2. Mechanism of Recognition of Cancer Cells by Immune System

Recent studies indicate that cancer cells are formed constantly in human organisms as a result of oncogenic activity of external as well as internal factors. However, such cells usually do not have a chance for an uncontrolled growth since they are quickly recognizable by the native immune system, and are then effectively eliminated by lymphatic phagocytic cells (so called *killer cells*) [4]. While the mechanism of “devouring” of the cells foreign to the organism is well recognized, very little is known about the way how they are protected from the immune diagnosis. After immune identification of “foreign” objects, which includes malignant abnormal cells, specialized white blood cells secrete active proteolytic enzymes that attack and dissolve such cells [5]. It turned out, however, that in the case of advanced human cancers, tumor cells are resistant to the destructive activity of lymphocytes, which do not eliminate them, but attack the surrounding healthy tissues of the organ. A classic example is prostate cancer, which is particularly resistant to chemotherapy, as well as immunotherapy induced by externally cultured and intravenously administered phagocytic cells [6].

**Figure 1:**
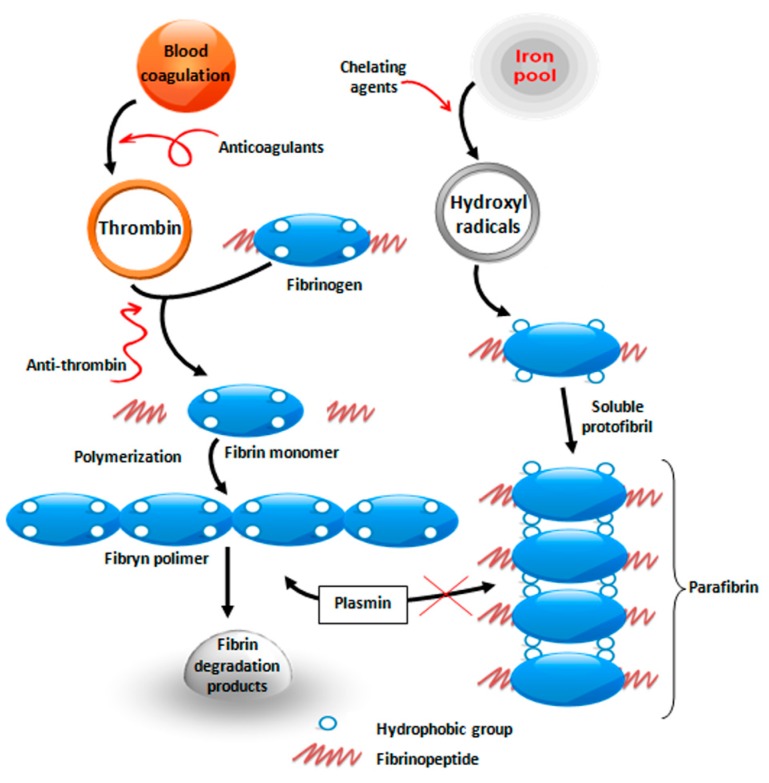
Schematic representation of thrombin-induced blood coagulation (left part) and the conversion of fibrinogen to insoluble polymer under the influence of iron (right part) based on references [7,8].

Prostate cancer cells are surrounded by a protective layer of a specific blood protein fibrinogen. This protein is highly soluble and is converted to insoluble fibrin only under the influence of thrombin, which is activated in case of vascular wall damage. A blood clot (fibrin) is formed this way, and it plugs the wound, thus preventing blood loss. Over time, the fibrin must be removed by proteolytic system (so called fibrinolysis) in order to make space for the growth of connective tissue cells and proper wound healing [3] (Figure 1).

It has been known for a long time that cell membranes of rapidly dividing cells, thus also cancer cells, are rich in free sulfhydryl groups (–SH), which are commonly found in the reducing environment of the cytoplasm [7]. For unknown reasons, the expression of sulfhydryls is observed on cancer cell membranes, which causes the exchange of disulfide between the polypeptide chains of fibrinogen that results in the formation of high-molecular polymer similar to fibrin, called parafibrin [8,9]. The difference between fibrin and parafibrin is that the latter is completely resistant to proteolytic degradation, and, therefore, forms a “shell” on the surface of tumor cells protecting them from destruction by phagocytic cells.

## 3. Unusual Properties of Selenium 

Selenium (Se), as well as sulfur (S), is an essential element that is naturally occurring mainly in two inorganic forms: as selenite (Se^4+^) and selenate (Se^6+^), and in a number of their organic derivatives. In contrast to sulfur, the concentration of selenium in soil is found to be very uneven, and as a result, their levels in agricultural products, mainly cereals, are also extremely differentiated, depending on the region in which they are cultivated [9,10,11]. Accordingly, the daily intake of selenium by humans varies widely between 50 µg and 600 µg. In some geographical areas, where the concentration of this element is found to be very low in the soil (e.g., Keshan region in China), people undergo pathological changes very commonly, for example, cancers and cardiovascular system diseases [12,13]. This extremely unbeneficial situation changed dramatically when the soil was enriched with selenite. This unusual relationship between the occurrence of cancer and the content of selenium in the diet was first demonstrated by the American researcher, G. N. Schrauzer [14], and this has recently been undertaken by the British biochemist Margaret Rayman [15]. Experimental and clinical studies demonstrated that selenium exhibits anticancer activity [1,9]. Selenium deficiency can affect the immune system by declining the development and functions of the thymus responsible for the production of macrophages and lymphocytes [7].

Selenium compounds exhibit different cytotoxic properties. The effect of selenium on cancers depends on its chemical forms, dose, cancer types, and the degree of selenium bioavailability. The effects of different selenium compounds on cancer cells—ranging from the most to the least—are in the following order: selenodiglutathione > selenite> selenocystine > selenate > hydrogen selenide > dimethylselenium > selenomethionine [16]. However, not all of the researchers confirm exactly such an order. The mechanisms by which selenium exhibits anticancer properties are not fully understood. A reverse relationship between selenium and neoplastic diseases such as cancers of alimentary tract, lung, or prostate, has been observed in clinical studies so far [17]. Recent clinical trials demonstrated that selenium yeast supplementation effectively reduced the incidence of prostate cancer by 60% [18]. In the case of colorectal and lung cancers, a decrease in total mortality of about 50% was observed [19]. Clinical studies suggest that supplementation of selenium (Se) in the form of selenium yeasts that mainly contain selenomethionine (SeMet) can reduce the risk of cancer in China and the USA [20]. In conclusion, it should be noted that selenium intake appears to be profitable not only in the prevention of cancer, but it can also positively affect many other functions in an organism by regulating blood pressure, reducing inflammation states, or preventing heart diseases [21]. In the global medical community, there is an unwavering belief that selenium and its compounds are toxic [22]. As the chemistry of this compound is poorly understood within this community, what forms of this element are toxic and beneficial are not properly recognized [23]. Understanding the effect of selenium on the prevention of cancer, and why it is effective in some cases and not in others, may shed new light on this issue. It should be primarily emphasized that the physicochemical and biological properties of selenium differ substantially depending on its valence, and thus selenite Se^4+^ exhibits an ability to undergo oxidation and reduction reactions (the so-called *redox reactions*), while selenate Se^6+^ is completely devoid of this ability. It obvious that such a small difference in the number of electrons in the outer orbital of selenium atom has a decisive effect on the physicochemical properties of these two forms of inorganic selenium. Thus, only Se^4+^ reacts with the –SH groups of proteins and prevents the formation of protein polymers that are rich in disulfides (parafibrin).

In conclusion, it should be noted that high doses of selenium generate oxygen free radicals, which in turn leads to an apoptotic cancer cell death inducing oxidation and crosslinking of sulfhydryl groups present in the proteins. The presence of an excess of reactive oxygen species indicates that cancer cells are often found in conditions of low oxygenation. As a result, the cancer cells are more prone to oxidative stress than the normal cells (their endogenous antioxidant systems are sufficient). This significant difference between normal and cancer cells can be potentially used for therapeutic purposes. Based on the these literature data [21,24], it should be emphasized that selenium exhibits advantageous anticancer properties and initiates apoptotic mechanism by participating in processes affecting changes in proteins conformation structure (signaling molecules, suppressor enzymes, and transcription factors) required for cell survival.

## 4. Prooxidative Effect of Selenite (Direct Anticancerogenic Effect)

The protective barrier on cancer cell membranes is made of blood proteins with fibrin-like properties, which, however, is not subject to the normal fibrinolytic degradation process. As a result, it permanently remains on such membrane and does not allow for its immune recognition as a “foreign” body. Moreover, the structure of such protein is not subject to degradation by proteolytic enzymes secreted by phagocytic cells of blood and leads to their further uncontrolled growth. Sodium selenite inhibits the disulfide exchange on the surface of cancer cell membranes, and thus makes it susceptible to destructive activity of phagocytic cells. The mechanism of sodium selenite interaction with sulfhydryl groups (–SH) of the cell membrane is explained in the following equation [3]:

P–[SH]_2_ + Se^4+^ → P–S–S–P + Se° (1)

where the sulfhydryl groups are converted to disulfide and selenite (Se^4+^) is reduced to elemental selenium (Se°) with a characteristic red color [25,26].

Cancer cells develop best under anaerobic conditions, which makes them particularly susceptible to the activity of oxidizing agent such as sodium selenite [27]. An increased rate of glycolysis enables the compensation for a small energy gain resulting from anaerobic respiration, which allows for cancer cells to continue the processes of uncontrolled growth and proliferation [28]. Therefore, cancer cells can develop continuously without any particular order.

Regarding the dosage of sodium selenite, it should be realized that previous data on this compound toxicity are inaccurate. According to current standards, it is assumed that the toxic effect of selenite starts from 600 μg per day, and transient toxic symptoms occur in the case of chronic dose higher than 1000 μg; nail fragility and hair loss are some examples [29].

Paskett et al. [30] demonstrated that sodium selenite is a promising non-toxic anti-inflammatory agent that spontaneously reduces lymphedema volume. In addition to the biological effects, sodium selenite also exhibits other pharmacological effects. The exact mechanism of this effect is unknown so far, but it is possible that this element affects the inhibition of adhesion proteins expression [30,31]. Zimmermann et al. [32] and Kasseroller [33] concluded that sodium selenite can reduce the incidence of recurrent subcutaneous tissue infections (erysipelas) with *Streptococcus pyogenes* after breast cancer treatment. Despite the promising results, further studies are needed to confirm the conclusions and to determine the optimal selenium dose and therapy duration [21].

The experimental data presented by Sinha and El-Bayoumy [34] demonstrated that selenium may affect the inductance of apoptosis process in various types of cancer cells, including prostate cancer, colon cancer, liver cancer, leukemia, and lymphoma. Selenium compounds exhibiting redox properties can produce a certain amount of reactive oxygen species (ROS). This ROS species has a prooxidative effect on cancer cell apoptosis [35]. Based on these data, it was suggested that this might be one of the mechanisms through which certain selenium compounds inhibit the development of cancers.

Oxidative stress has been found in cancer cells, but the mechanisms responsible for its induction are not fully explained. High concentrations of ROS in cancer cells may be responsible for the rapid rate of cell division. Furthermore, increased production of reactive oxygen species (ROS) may exhibits a high cytotoxic effect on cancer cells proliferation as compared to normal cells [36]. Therefore, redox-active selenium compounds producing reactive oxygen species are a new class of therapeutic agents that target an uncontrolled redox homeostasis of cancer cells. Significant oxidative changes due to high RFT concentrations may eventually lead to cell necrosis. It should be emphasized that the use of high doses of sodium selenite exhibits promising anticancer effects, as described in numerous preclinical studies [37]. Numerous studies demonstrated higher selenite cytotoxicity against cancer cells when compared to normal cells, using a comparable dose of this element [38].

The study conducted by the Pomeranian Medical University in Szczecin demonstrated that selenium level in serum is related to an increased risk of cancer diseases. The study evaluated selenium levels in blood serum of 86 patients with lung cancer and 86 healthy ones. The results demonstrated a strong correlation between selenium content and cancer. In the case of lung cancer, the average selenium content was about 63.2 μg/L, whereas for the control group, the average level was 74.6 μg/L. Also, the risk of lung cancer was tenfold lower in patients whose selenium concentration in serum was 80 μg/L [14].

Similar data were presented by Lener et al. [39]. The authors evaluated the risk of getting colon cancer in patients whose serum content of selenium was <40 μg/L when compared to patients whose serum level was >72 μg/L.

Xiang et al. [40] demonstrated that sodium selenite can cause cell death by an independent pathway of mitochondrial apoptosis, endoplasmic reticulum stress (caused by the presence of (non)unfolded proteins), processes of autophagy, or necrosis. Cancer cell death can be induced by high concentrations of selenium (50 μM and 100 μM) [18] than only at apoptotic levels of this element, suggesting that the processes of cancer cells inactivation induced by selenite may involve other mechanisms [40].

## 5. Selenite as a Substrate for Synthesis of Selenoproteins with Antioxidant Properties

Selenium (Se) exists in a human organism in various organic forms, mostly as selenoproteins, the biosynthesis of which utilizes inorganic forms of selenium [11]. Selenium can be considered as a mineral necessary for the proper functioning of living organisms [1]. The most important biological role of selenium is associated with its presence in active centers of many enzymes and proteins, as well as with its antioxidative role. Selenium activates anticancer agents, prevents heart and vascular diseases, exhibits anti-proliferative and anti-inflammatory properties, and stimulates the immune system [2]. The biological role of selenium is based on the prevention of infertility and cancer and cardiovascular diseases [41]. Selenium constitutes an integral part of selenium-containing proteins and several antioxidant enzymes, such as glutathione peroxidase (GPx), thioredoxin reductase (TRxR), and iodothyronine deiodinase (DIO), which protect cells from the harmful effects of free radicals formed during oxidation [42].

Although it is well documented that Se deficiency is associated with an increased incidence of cancer morbidity and mortality, not much is known about its anticancer mechanism of action. It is generally believed that Se compounds have antioxidant properties that are responsible for the control of cancer growth and spread. Not all forms of organic Se are equally effective, only those with oxidant activity can have anticancer property [5].

Tumor cells express free sulfhydryl groups (–SH) on their cell membranes and contribute to their uncontrolled cell division. Thus, it is obvious that only those compounds that can oxidize these groups to disulfides (S–S) may inhibit this process. While some organic forms of Se, such as selenocystine, methylseleninic acid, and Se-methylselenocysteine are known to be antioxidants, their anticancer effect is still not well understood [43]. Certainly, they cannot react with free sulfhydryls, because their Se moiety is already involved in the covalent bonds with sulfur atom in these compounds. Although it is possible that in the in vivo situation organic forms of Se may reductively release inorganic forms of selenium, specifically selenite that is able to oxidize sulfhydryls to disulfides. This mechanism has been implicated in the formation of a protective protein coat on tumor cell membranes, thus making them unrecognizable by the innate immune system [29,37].

One of the marginal problems of selenium use is its narrow range between the toxic dose and the dose necessary for the proper functioning of living organisms. Sodium selenite may be toxic when taken orally at higher doses, yet it is well tolerated by other routes such as intravenous, intraperitoneal and/or transdermal. Although the exact mechanism of oxidative imbalance in cancer is not completely understood, the current state of knowledge indicates that sodium selenite may be an ultimate remedy in the treatment of cancer. Future studies should concentrate on the selection of those types of cancer that are most sensitive to the action the redox-active forms of selenium capable of scavenging of hydroxy free radicals. Numerous clinical and experimental studies demonstrated that specific selenium compounds (e.g., SeMet, MSA, SeCys, and SeO_3_^2−^) have different antioxidant activity, which suggests that each compound must be considered individually, depending on its individual antioxidant properties [21]. The protective effect of selenium against the development of cancer diseases is related to the activity of hydrogen selenide and selenomethionine present in the cells. These compounds may be responsible for the modification of protein thiols, resulting in an increased efficiency of RNA methylation [17]. Moreover, organic and inorganic forms of this element induce the activity of *p53* gene toward DNA repair or apoptosis.

As demonstrated recently by Swedish scientists, considerably higher doses of selenium are well tolerated by patients with cancer, in the case when sodium selenite is administered intravenously. It should be emphasized that these types of clinical trials conducted at the Karolinska Institute in Sweden are one of the first trials in the world [37].

The above-mentioned studies demonstrate an excellent activity of sodium selenite in the prevention of cancers and clearly demonstrate the unique properties of this element as compared to other antioxidants. However, the mechanisms of how selenium inhibits the proliferation of cancer development are not fully understood. 

## 6. Conclusions

Selenium compounds are usually cheap, and when given in the right dose, they are harmless to organisms. Therefore, supplementation of this element is an attractive possibility to reduce the incidence of cancers in many groups of people around the world. Despite the availability of huge data about the beneficial effects of sodium selenite, the question arises as to why it is not available as a drug. Firstly, it is not possible to obtain a patent on this so simple and cheap compound. Secondly, a key argument against the use of untested substances in cancer treatment exists. However, this argument is totally false, since it concerns only those diseases that need life-saving medicines (e.g., antibiotics in infectious diseases). In case of cancer, there are no such means that eliminate cancer, but only extend the life of the patient. Therefore, intake of foreign substances does not threaten human life, as is the case of infections, in which administration of an inappropriate antibiotic usually ends up in the death of the patient. In the case of sodium selenite, that argument it is completely inadequate in light of the huge database existing in the world literature. It seems that the most reasonable approach would be to cooperate with medical centers such as those in Sweden, which have already successfully used sodium selenite in the treatment of cancer. Although it is unlikely, governmental or public institutions, which would introduce such initiatives, should be established. To do this, there is a need for thorough changes in the legislature aimed at protecting the life and health of patients with cancer. Last but not least, is the possible therapeutic effect of anticoagulation coupled with the removal of iron from the blood of cancer patients [44].
